# A spectrogram image based intelligent technique for automatic detection of autism spectrum disorder from EEG

**DOI:** 10.1371/journal.pone.0253094

**Published:** 2021-06-25

**Authors:** Md. Nurul Ahad Tawhid, Siuly Siuly, Hua Wang, Frank Whittaker, Kate Wang, Yanchun Zhang

**Affiliations:** 1 Institute for Sustainable Industries & Liveable Cities, Victoria University, Melbourne, Victoria, Australia; 2 Nexus eCare, Adelaide, South Australia, Australia; 3 School of Health and Biomedical Sciences, RMIT University, Melbourne, Victoria, Australia; National University of Sciences and Technology, PAKISTAN

## Abstract

Autism spectrum disorder (ASD) is a developmental disability characterized by persistent impairments in social interaction, speech and nonverbal communication, and restricted or repetitive behaviors. Currently Electroencephalography (EEG) is the most popular tool to inspect the existence of neurological disorders like autism biomarkers due to its low setup cost, high temporal resolution and wide availability. Generally, EEG recordings produce vast amount of data with dynamic behavior, which are visually analyzed by professional clinician to detect autism. It is laborious, expensive, subjective, error prone and has reliability issue. Therefor this study intends to develop an efficient diagnostic framework based on time-frequency spectrogram images of EEG signals to automatically identify ASD. In the proposed system, primarily, the raw EEG signals are pre-processed using re-referencing, filtering and normalization. Then, Short-Time Fourier Transform is used to transform the pre-processed signals into two-dimensional spectrogram images. Afterward those images are evaluated by machine learning (ML) and deep learning (DL) models, separately. In the ML process, textural features are extracted, and significant features are selected using principal component analysis, and feed them to six different ML classifiers for classification. In the DL process, three different convolutional neural network models are tested. The proposed DL based model achieves higher accuracy (99.15%) compared to the ML based model (95.25%) on an ASD EEG dataset and also outperforms existing methods. The findings of this study suggest that the DL based structure could discover important biomarkers for efficient and automatic diagnosis of ASD from EEG and may assist to develop computer-aided diagnosis system.

## Introduction

Autism spectrum disorder (ASD) is a group of complex neurological developmental disorders that includes autism, childhood disintegrative disorder, Asperger’s syndrome and an undetermined form of pervasive developmental disorder [1]. The range and severity of ASD symptoms can vary widely that commonly includes difficulty with social communication and interactions, obsessive interests, reduced eye contact and restricted or repetitive behaviors. ASDs begin in early childhood, mostly within five years of age and remains for the rest of the life [2]. World Health Organization (WHO) reported that globally in every 160 children, one child diagnosed as ASD [2]. According to the autism spectrum, autism frequency rate increases around 40% from one in 100 to an approximate one in 70 in Australia [3]. The Centers for Disease Control and Prevention (CDC) stated that one child in about 54 children is diagnosed with an ASD in the U.S. in 2020 [4]. ASDs can substantially restrict an individual’s ability to carry out everyday activities and participate in social movement. ASDs often negatively impact the person’s educational and social achievements, employment opportunities, limit the capacity to conduct daily activities and participation in society. Globally, ASD individuals are frequently subjected to abuse, differentiation and infringement of human rights. [2]. So far, no cure has been found for ASD but early intervention can improve brain development and also can enhance learning, communication and social skills. To do so, an effective, efficient and high accuracy system is needed to diagnose ASD.

The human brain with about 86 billion neurons, is considered to be the most complicated biological system in the known world that regulates all our thoughts, perceptions, memories, feelings and actions. Functional condition of the brain is a big data collection and also the main source of information for neurological disorder diagnosis which makes it a vital and wide area of study in the field of biomedical science. Different techniques are available for capturing the functional activity of the brain such as, positron emission tomography (PET), magnetic resonance imaging (MRI), functional magnetic resonance imaging (fMRI), electrocorticography (ECoG) and electroencephalography (EEG) [5–7]. Among these techniques, EEG is widely used because of its outstanding temporal resolution, usability, non-invasiveness, low set-up costs and mass availability for clinicians [8]. Neurons uses electrical impulse of different frequency bands for their communication which is recorded in EEG through electrodes attached to the scalp. This produces a large volume of EEG multi-channel signals that neurologists visually interpret to identify and recognize neurological disorders [9]. However, due to the lack of standard assessment criteria, this visual inspection is not a rational evaluation technique and also time-consuming, error-prone, exhaustive, subjective decision with human error and has reliability problem [10].

As the technological advancement grows day by day, so the computer-aided diagnosis (CAD) has become an integral part of the medical industry. Various studies have carried out to diagnose ASD using EEG signal. Those studies can be broadly divided into two groups depending on the feature extraction and classification technique. First approach is known as machine learning (ML) technique in which different time-frequency based features are extracted from EEG signal and then those extracted features are used for ASD classification using different ML based classification techniques. The key success of the ML based classification depends on mining significant features from EEG signals and till now various researchers have attempted this approach for ASD classification. Sheikhani *et al*. [11] used short-term Fourier transform (STFT) for feature extraction and *k*-nearest neighbor (*k*NN) for classification to achieve an accuracy of 82.4% over a dataset consists of 17 subjects (10 ASD and 7 control group). In a later study [12], they used STFT and statistical analysis with *k*NN on a dataset of 28 subject (17 ASD, 11 control group) to achieve 96.4% accuracy. Bosl *et al*. [13] proposed a diagnostic approach to use EEG data as a biomarker for children at a high risk for ASD. They extracted features using minimum mean square error (mMSE) and classified using *k*NN, naïve bayes (NB), and support vector machine (SVM) to produce classification accuracy over 90% on a dataset of 79 infants (46 high risk for autism (HRA), 33 controls) of 6 to 24 months of age. In a later study [14], they used a data-driven approach for ASD classification in which EEG data from 188 infant (89 low-risk controls (LRC), 99 HRA; ages of 3 to 36 months) participants were used. EEG signal was decomposed into six sub bands using wavelet transform (WT) and nine different non liner features were extracted from each sub bands. Using leave-one-out cross-validation, they evaluated obtained features as input to SVM for classification and achieved a sensitivity and specificity value exceeding 95% at some ages in distinguishing ASD subjects from the LRC subjects. In another study, Eldridge *et al*. [15] used variance in time by computing the sum of signed differences (SSD) and mMSE features from pre-processed signals and fed to different classifiers. On a dataset of 49 children (19 ASD and 30 Non ASD), highest accuracy of 79% was achieved with NB classifier. In [16], Grossi *et al*. introduced a complex algorithm named MSROM/I-FAST for EEG processing with seven machine learning algorithms namely: sine net neural networks (Sn), logistic regression (LR), sequential minimal optimization (SMO), *k*NN, K-contractive map (K-CM), NB and random forest (RF) to classify autism. Using 25 subjects’ (15 ASD and 10 typically developing (TD)) resting state EEG data with leave-one-out cross-validation a highest accuracy of 92.8% was achieved with RF classifier. Heunis *et al*. [17] used recurrence quantification analysis (RQA) for feature extraction from resting state EEG of 14 children (7 ASD and 7 TD) and feed into SVM classifier. They yielded a high accuracy of 92.9% with leave-one-subject-out validation process. A decision support system (DSS) named ASDGenus was proposed by Haputhanthri *et al*. [18] for ASD diagnosis using 15 participants (10 ASD, 5 control) EEG data. They combined statistical features (mean, standard deviation) before and after applying DWT for each channel and applied correlation-based feature selection (CFS) for significant feature selection. Finally, four classifiers (LR, SVM, NB and RF) were applied and the highest achieved accuracy was 93% using RF classifier. In a later study [19], they extended ASDGenus incorporating shannon entropy for feature extraction of the EEG data and also used the average temperature of the thermogram face images of the participants. Using a dataset of 17 participants (8 ASD, 9 control), they achieved an accuracy of 88% using only EEG data with RF classifier and 94% accuracy using both EEG and thermogram image data with both LR and MLP classifiers. In a recent study [20], abdolzadegan *et al*. extracted linear (power spectrum, wavelet transform, fast fourier transform) and nonlinear (fractal dimension, correlation dimension, lyapunov exponent, entropy, detrended fluctuation analysis and synchronization likelihood) from 45 subjects’ (34 ASD, 11 non ASD) EEG data and applied different feature selection techniques (mutual information, information gain, minimum-redundancy maximum-relevancy and genetic algorithm). For classification SVM and *k*NN used and achieved an accuracy of 90.57% and 72.77% respectively. Functional connectivity and temporal relationship of brain activity was used to classify and predict the severity score of ASD by jayarathna et al. [21]. They decomposed the signal into five feature bands (delta, theta, alpha, beta, and gamma) and five feature sets were created using amplitude and power for each electrode. Using 43 different classifiers on a dataset of 17 subjects (8 ASD and 9 control), they achieved an accuracy of 98.06% using JRip. In the ML based classification approaches mentioned above build features from raw EEG data using feature engineering approach, which needs experts with comprehensive knowledge of the target feature domain [22].

Recently, deep learning (DL) based classification approaches are getting popular among the researchers due to its ability to learn features from raw data automatically and also perform classification using those features in automatic process [22]. Ahmadlou *et al*. [23] proposed a fractality and wavelet-chaos-neural network based ASD diagnosis system. They introduced the idea of using fractal dimensions (FDs), a non-integer dimension that shows the degree of complexity and self-similarity of a signal as a feature. Using Eye-closed EEG data from 17 subjects (9 ASD, 8 TD) with a two layer radial basis function neural network (RBFNN), they achieved 90% accuracy. Using the same database in their later study [24] where they used improved visibility graph (VG) for fractality investigation based feature named power of scale-freeness of VG (PSVG). Enhanced probabilistic neural network (EPNN) was used for classification and got an accuracy of 95.5%. In another study of same authors used the analysis of functional connectivity of brain using fuzzy Synchronization Likelihood and diagnoses ASD based on that [25]. Using EEG data from 18 subjects (9 ASD, 9 TD) with EPNN classifier they obtained 95.5% accuracy. Djemal *et al*. [26] introduced an ASD diagnosis system using discrete wavelet transform (DWT), shannon entropy (SE) and artificial neural network (ANN). Two-layer ANN with ten-fold cross-validation was used for classification on an EEG dataset of 19 subjects (9 ASD and 10 non ASD). They segmented the data into 50 second long segments with two different overlapping techniques. With half overlapping segmentation provided very good classification accuracy of 99.7% and without any overlapping in segmentation got an accuracy of 98.6%. Alturki *et al*. [27] also used 50 second segmentation and decomposed the signal into sub bands using DWT. They used logarithmic band power (LgBP), standard deviation (SD), variance, kurtosis, and SE to extract features from the segmented decomposed sub bands and feed those features to different classifiers namely: linear discriminant analysis (LDA), SVM, *k*NN, and ANN. Using a dataset of 19 children (9 ASD and 10 non ASD), they achieved the highest accuracy of 98.2% using SE and ANN classifier. The main benefit of the DL based classification approaches is that it does not require any specific feature domain experts for feature extraction from the raw data. The DL methods perform both feature extraction and classification automatically and generates better results than ML based classification processes but it works as a black box to the user [28]. In the same study [21], the authors have also used CNN model on power spectrum of electrodes to evaluate the long-term dependencies between ASD and EEG data. Using a three layer CNN model they achieved an accuracy of 90%. Sometimes researchers use crafted features as input for DL models instead of using large scale raw EEG data to reduce the computation time and also for training the model with important features [23–27].

Although the state-of-the-art techniques have achieved various levels of effectiveness in some cases but their ability of robustness and generalization are limited. This is because it is often very hard to extract representative features from EEG signals using current techniques due to the non-stationary nature and presence of noise in EEG signals. In order to describe EEG, none of the previous approaches considered a two-dimensional (2D) time-frequency image where extraordinary features would be unveiled from different perspectives.

This study proposes a new 2D EEG spectrogram image-based scheme involving ML and DL for automatic identification of ASD. In this study, 2D time-frequency (T-F) spectrogram images are used for describing the nonstationary characteristics of EEG signals and finally the images are assessed through ML and DL based process, separately. For EEG signals, spectrogram image is a type of visual feature representation in the T-F domain. Frequency band varies with time in the spectrogram image, and various colors in the image reflect different energy values in the EEG signal. [29]. Previously, few studies have used time-frequency (T-F) based images for the classification of neurological disorders such as epilepsy [10], epileptic seizures [30], clinical brain death diagnosis [29], schizophrenia [28] and classification of sleep stage [31], but never for the classification of ASD. In our recent work [32], we have used this T-F based spectrogram image of EEG signal for ASD classification using ternary CENTRIST (tCENTRIST) [33] with SVM classifier. In this study, we have extended our previous work of T-F image-based ASD classification using both ML and DL based approach. In the ML based classification, we have used tCENTRIST for feature extraction from spectrogram images due to its computational simplicity and better performance. Extracted features are classified using six different classifiers named: NB, LDA, RF, *k*NN, LR and SVM. Ten-fold cross validation is used to evaluate the performance of ML based classification approaches. For the DL based scheme, three different convolutional neural network (CNN) models are used to classify the spectrogram images with the dataset divided into 70%, 15%, 15% for training, validation and testing the model respectively. The findings obtained from the proposed methods are contrasted with other current literature that used the same dataset.

This study’s major contributions are summed up as follows:

1. Introduction of an efficient and effective automatic classification model for identifying ASD from non ASD subjects using T-F spectrogram image of EEG signals.
2. Investigating the performance of six ML based classifiers with tCENTRIST based textual feature extraction method.
3. Investigating three different CNN models for DL based classification.
4. Improving the efficiency of classifications relative to existing methods.

The rest of this article is structured as follows. Section presents details of the proposed method. Section describes the dataset and performance evaluation parameters used in this study. Experimental results and their corresponding discussions are given in Section and finally, Section contains concluding statement and reveals possible future work.

## Methodology

**Fig 1:**
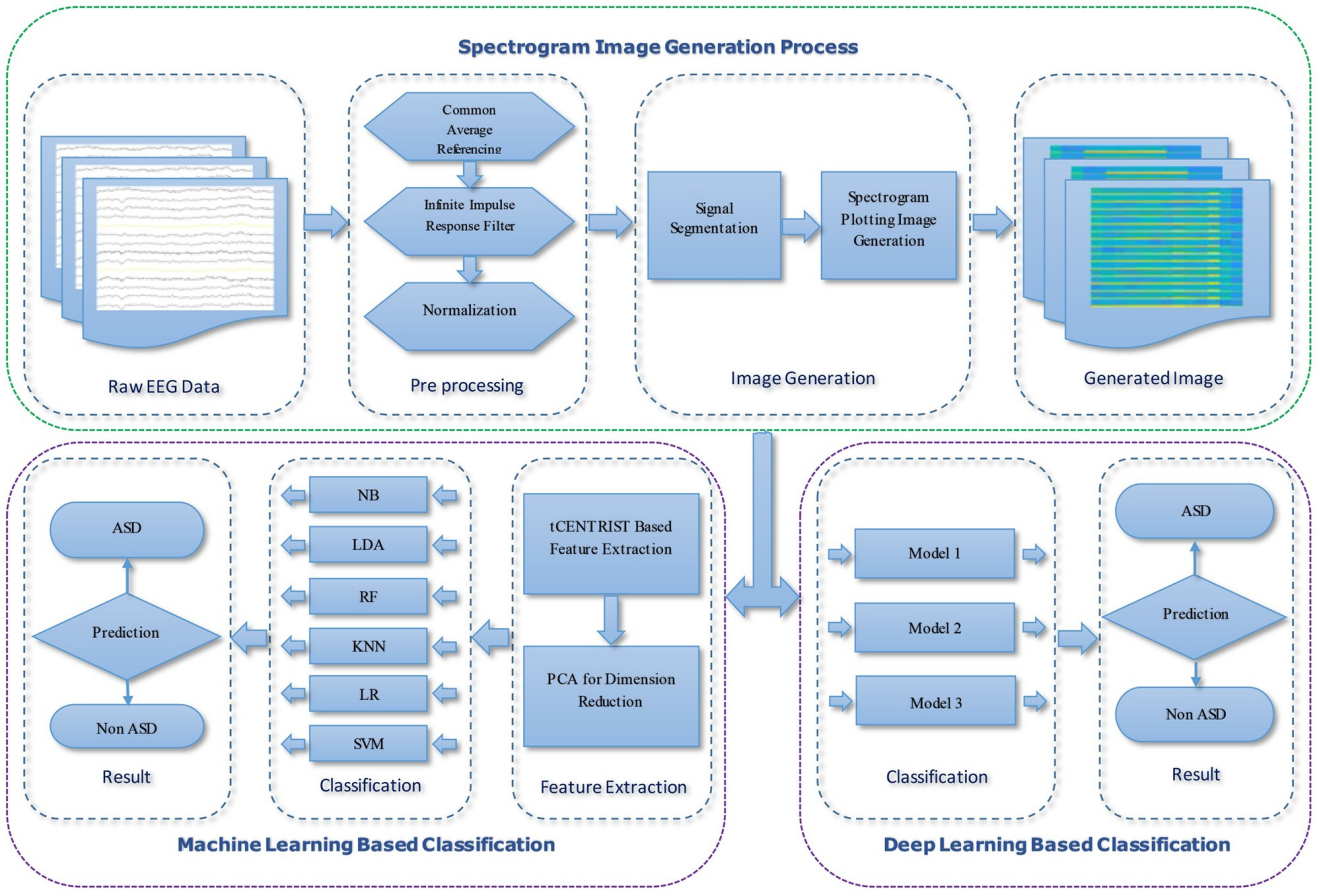
Overview of the proposed framework.

In this study, an ASD classification technique is proposed based on spectrogram image of EEG signals. Fig 1 provides a summary of the proposed framework. The system can be divided into three parts: pre-processing and spectrogram image generation, machine learning based classification and deep learning based classification.

### Pre-processing and spectrogram image generation

In this section, at first, artifacts are removed from raw EEG data using pre-processing techniques like re-referencing, filtering and normalization. Common average referencing (CAR) is used for re-referencing, in which the average value of all electrode collection (common average) is used as reference. In the second step of pre-processing, infinite impulse response (IIR) filter is used to low pass filter the signal at 40Hz cut off frequency and finally the filtered signals from each electrode is normalized to the interval [-1, 1]. After that, pre-processed signals are segmented into 3.5 second window frames for each subject of the dataset. Using Short-Time Fourier Transform (STFT) for each of the above segments, the spectrogram plot is generated in the last step and saved as image. These spectrogram images are used as input for both the ML and DL based methods.

### Machine learning based process

The ML based classification process consists of three sub processes: feature extraction, dimension reduction and classification. For extracting representative features from those spectrogram images, Ternary CENTRIST (tCENTRIST) is used. It is a Local Ternary Pattern (LTP) and CENsus TRanformed hISTogram (CENTRIST) based image feature extraction method proposed by Dey *et al*. [33]. Ternary CENTRIST (tCENTRIST) is a fusion of LTP and CENTRIST that uses LTP in place of Linear Binary Pattern (LBP) of CENTRIST [34]. This tCENTRIST technique has given better performance for garments texture classification [33] and gender classification from face image [35] and also computationally simple. It uses the Spatial Pyramid Matching (SPM) scheme to capture the global image structure that divides an image into sub regions and LTP based histogram of those regions are generated and concatenated to construct a single histogram as a feature for that image. Principal Component Analysis (PCA) is applied to reduce the extracted feature dimensions and finally the reduced features set is used as input to different ML based classifiers. For classification, six different classifiers are used: NB, LDA with pseudolinear discriminant analysis type, RF, *k*NN with nine as neighbor number (*k* = 9), LR and SVM with linear kernel (used LibSVM [36]).

### Deep learning based process

**Fig 2:**
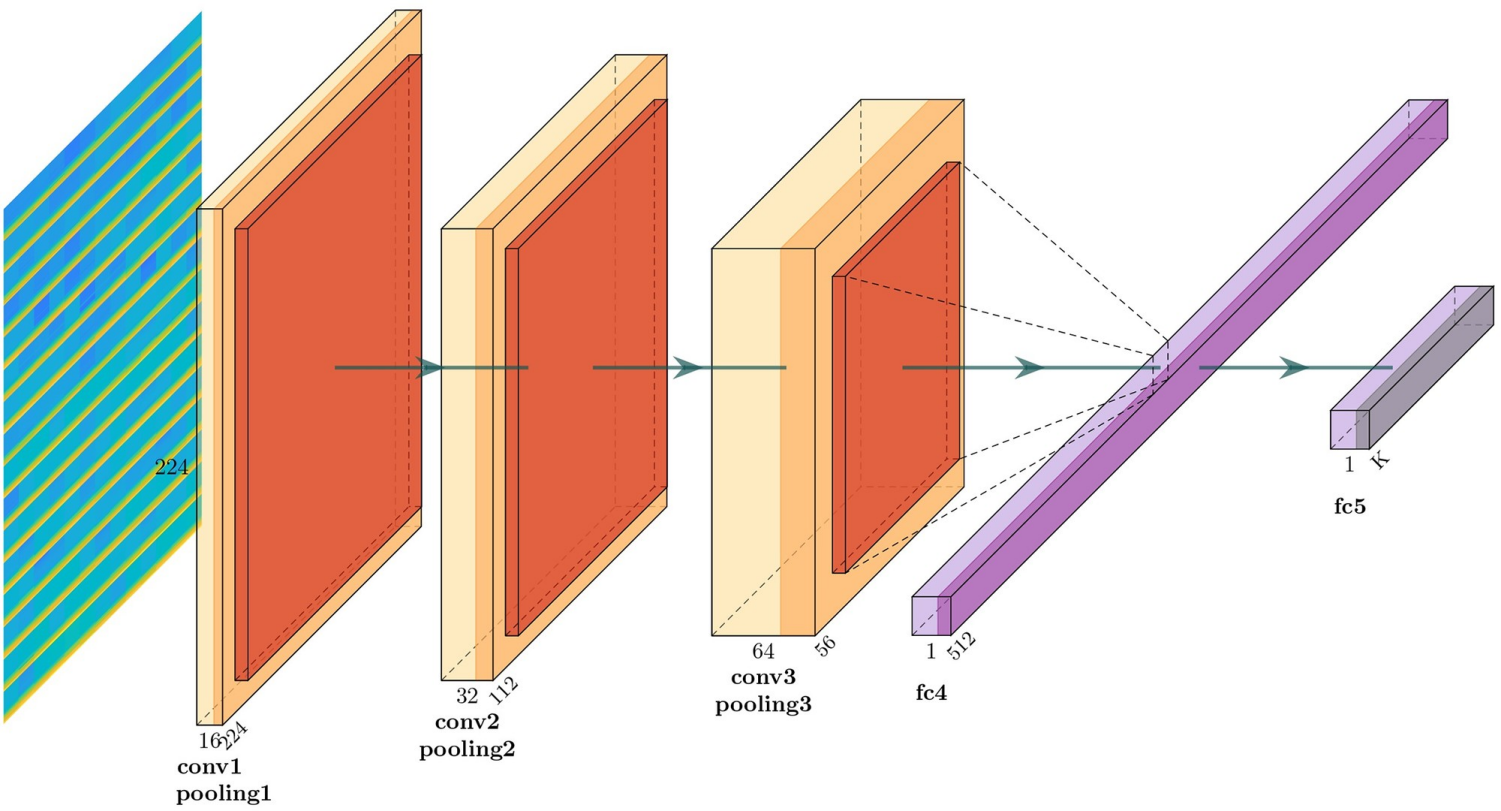
CNN model 1.

**Fig 3:**
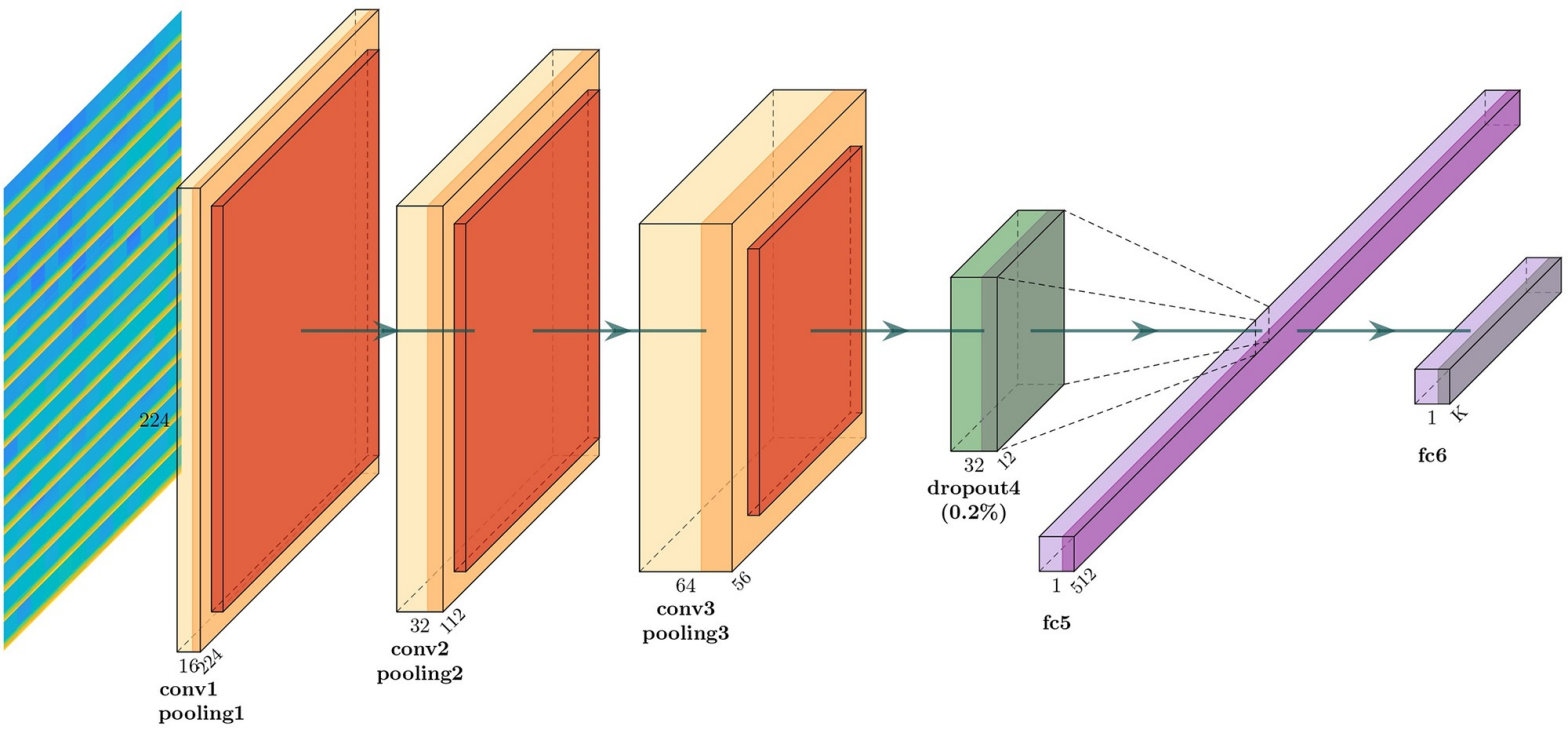
CNN model 2.

**Fig 4:**
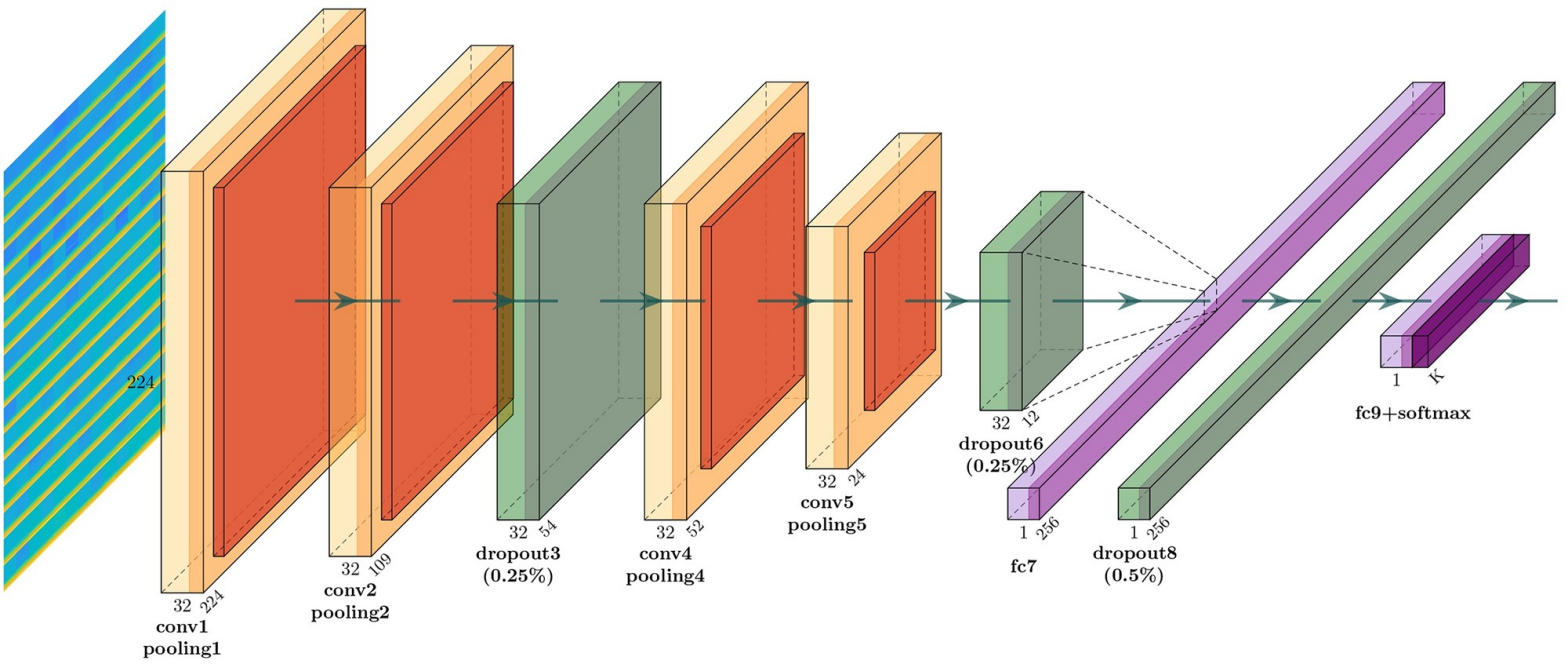
CNN model 3.

For the DL based classification, CNN model is used as it is the best DL model to deal with image related problems [37, 38]. In this study, three different CNN model is used for performance evaluation. Model 1 consists of three convolution blocks with each of them having a max pool layer. On top of it there is a fully connected layer with 512 units which is activated by a relu activation function. Overall layout of the model 1 is given in Fig 2. Second model is same as the first model except that 20% dropout is applied on the last max pool layer. Applying dropout will randomly set 20% of the neurons to zero during each training epoch. Fig 3 shows the block diagram of model 2. Last CNN model contains 4 alternating convolutional and max pooling layers, followed by a 25% dropout after every two convolution/max pooling pair. After the last pooling layer, a fully-connected layer with 256 neurons, then another 50% dropout layer, and finally a softmax classification layer for two classes is attached. Full layout of the model 3 is given in Fig 4.

## Performance evaluation

This section initially describes the detailed overview and pre-processing method of the dataset used in this experiment. After that, performance parameters with equations are discussed that are used for evaluating the performance of the proposed system.

### Dataset

**Fig 5:**
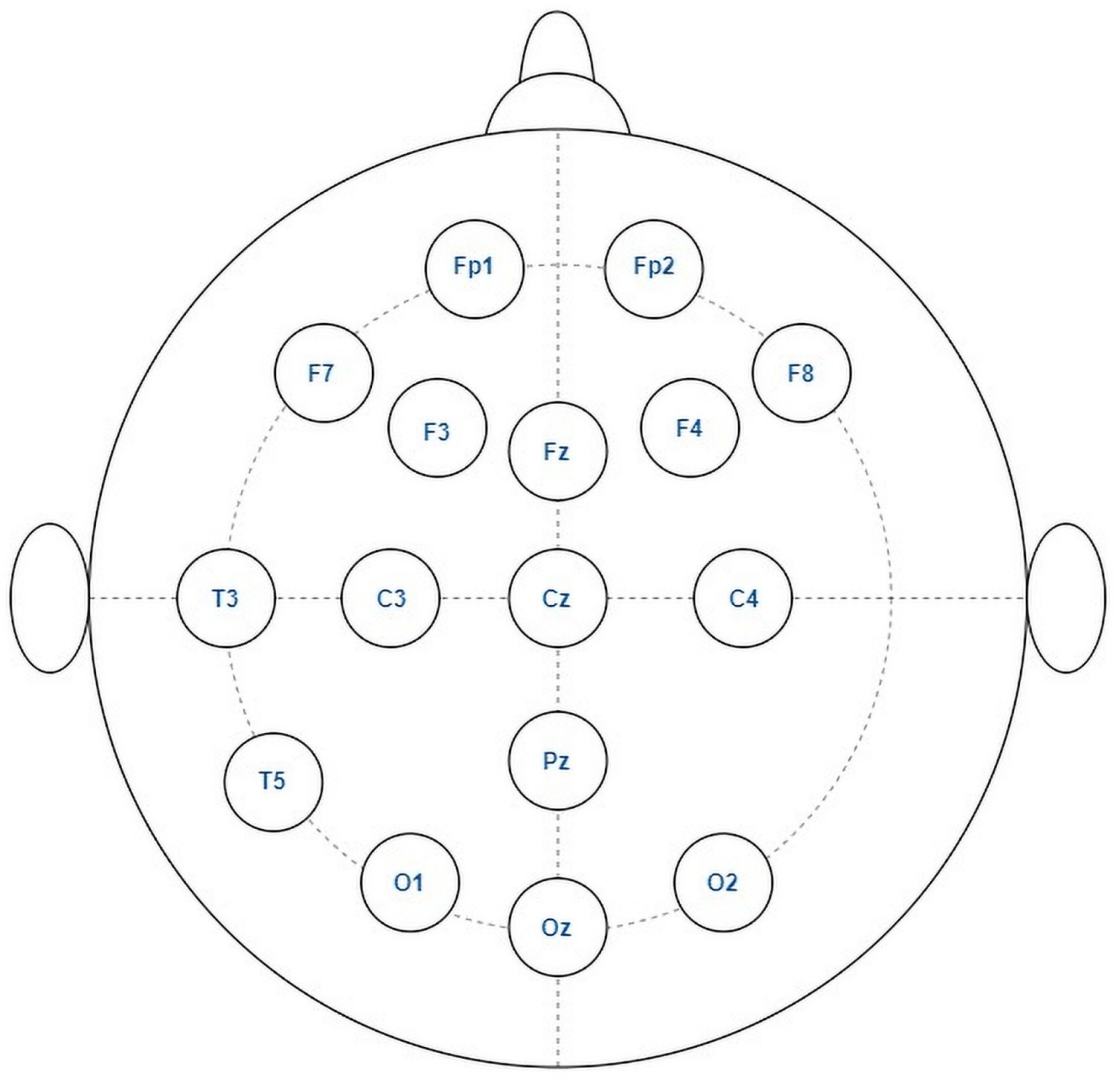
Electrodes placement of autism data acquisition system by [39].

This proposed method is evaluated using the dataset from King Abdulaziz University (KAU) Hospital, Saudi Arabia, Jeddah [39]. It is a publicly available dataset that can be found in this link, https://malhaddad.kau.edu.sa/Pages-BCI-Datasets.aspx. Anyone can access the dataset for their study through formal email request to the owner of the dataset Dr. Mohammed Jaffer Alhaddad (malhaddad@kau.edu.sa). We also did the same process and obtained the dataset for this study. Participants anonymity is ensured by not publishing any personal identification information of the subjects. The dataset contains sixteen subjects with twelve from ASD group (3 girls and 9 boys, age 6–20 years of age) and four subjects from control group (all boys, 9–13 years of age) with no past track of neurological disorders. Signals from subjects were recorded using Ag/AgCl electrodes with g.tec EEG cap, g.tec USB amplifiers, and BCI2000 software in relaxing state to get the artifact-free EEG data. The data recording was carried out from 16 channels using the international 10–20 system, as shown in Fig 5 with right ear lobe as REF and AFz as GND. A band-pass filter with a pass frequency band (0.1–60Hz) and a notch filter with a frequency band (60Hz) was used during the recording time to filter the dataset. Finally, all EEG signals were digitized at 256Hz sampling rate.

**Fig 6:**
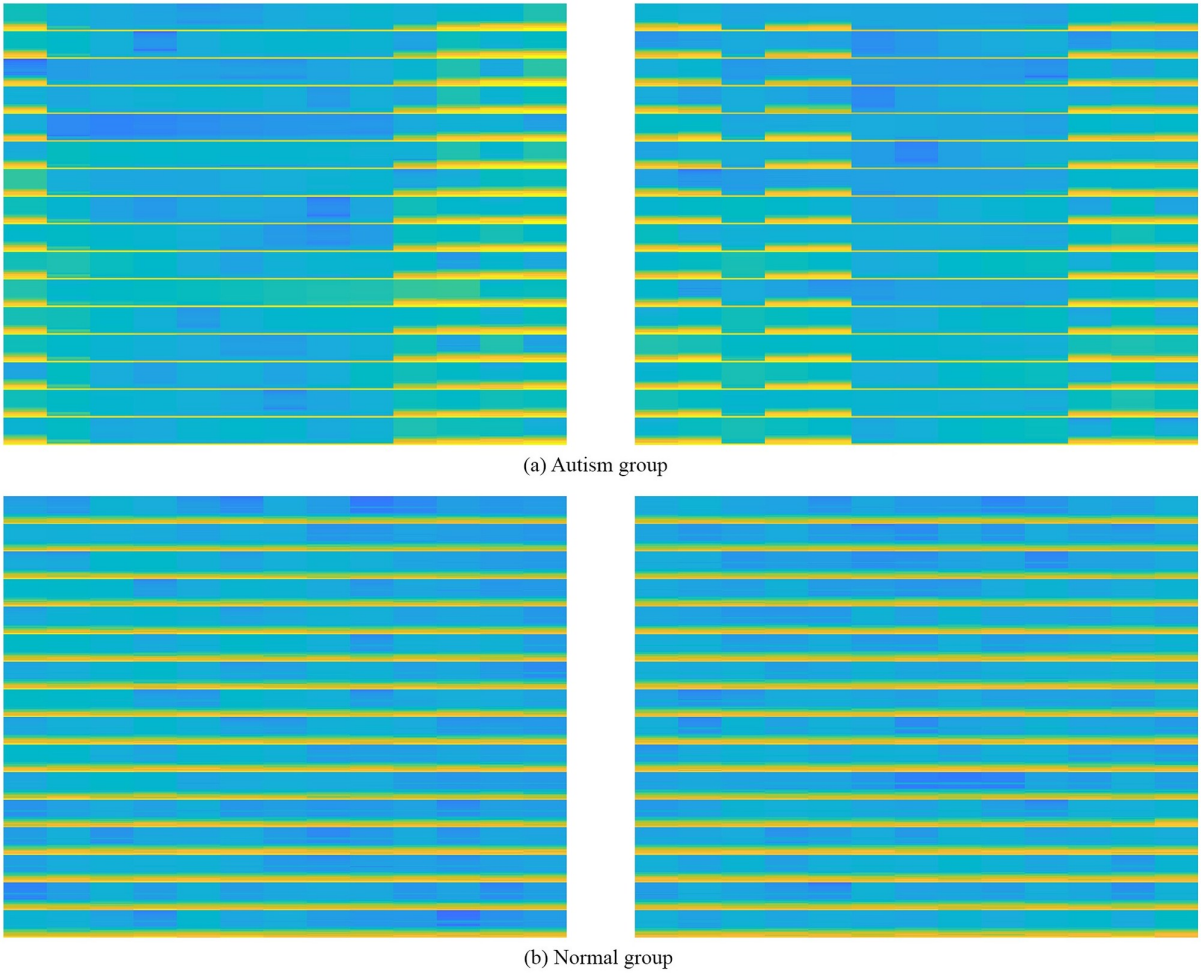
Sample spectrogram images. **(a)** Images from ASD group, **(b)** Images from non ASD subjects.

According to the proposed method, data is pre-processed using CAR, IIR and normalized. After pre-processing, those signals are segmented into 3.5 second time frames to get the precise information and finally spectrogram plot images are produced from those fragments using STFT. A total of 4657 images are produced with 3276 images from ASD subjects and 1381 images from non ASD subjects. Sample spectrogram images from the proposed method are given in Fig 6 where Fig 6a shows images from ASD group and Fig 6b shows images from non ASD subjects. These images are used as input for classification process of both ML and DL based techniques.

Image generation and ML based experiments are carried out in MATLAB (R2020a) environment in a computer with intel core i5 64bit processor with a frequency of 1.7GHz and 8 GB memory. DL based experiments are carried out in Google Colab environment [40].

### Performance evaluation parameters

The categorization performance of the proposed framework is measured using Receiver Operating Characteristic (ROC) parameters such as True Positive (TP), True Negative (TN), False Positive (FP), and False Negative (FN) to calculate the sensitivity, specificity, F1 score and overall accuracy using (1)–(4). These assessment criteria allow to estimate the performance behavior of the classifiers [41–46].

$$\begin{array}{c} Sensitivity=\frac{TP}{TP+FN}*100 \end{array}$$ (1)

$$\begin{array}{c} Specificity=\frac{TN}{TN+FP}*100 \end{array}$$ (2)

$$\begin{array}{c} Accuracy=\frac{TP+TN}{TP+FP+TN+FN}*100 \end{array}$$ (3)

$$\begin{array}{c} F1score=\frac{2\;TP}{2\;TP+FP+FN} \end{array}$$ (4)

Here,

- TP implies that spectrogram image from ASD subject is correctly diagnosed as ASD class.
- TN implies that spectrogram image from healthy subject is correctly diagnosed as healthy class.
- FP implies that spectrogram image from healthy subject is falsely diagnosed as ASD class.
- FN implies that spectrogram image from ASD subject is falsely diagnosed as healthy class.

ROC graph is a very useful tool to visualize the reliability of the classifier which is generated by plotting sensitivity (true positive rate) in Y-axis and 1-specificity (false positive rates) in X-axis. Area under the ROC curve (AUC) is a common metric for evaluating the performance of binary classifier. AUC value always maintains the below inequalities:

$$\begin{array}{c} 0\leq AUC\leq 1 \end{array}$$ (5)

It is evident from 5 that the AUC value 1 indicates the classifier has a perfect capacity to discriminate and the value below or equal to 0.5 implies that the classifier has no discriminative ability at all [47].

## Result and discussion

In this section, detailed results of the proposed classification methods are discussed. This discussion is done in two subsections for the proposed two different classification process: ML based classification performance and DL based classification performance.

### Results for machine learning based process

For ML based classification, performance of the proposed system is evaluated using *k*-fold cross-validation technique. In this process, dataset is arbitrarily divided into *k* subsets of equal size. Then the performance is evaluated by training the system using *k-1* subsets and testing is done by using the remaining subset. This process is iterated for *k* times (*k*-fold), where each subset is used once for testing. Here, ten-fold cross-validation is used, where spectrogram images are divided into two subsets of 90% and 10% for training and testing respectively. This process is repeated for 10 times so that every image from the dataset should belong to the test subset exactly once. Finally, the results from the ten-fold are averaged to produce a single overall classification metrics. In ML based classification, six different classifiers are used: NB, LDA with pseudolinear discriminant analysis type, RF, *k*NN with *k* = 9, LR and SVM with linear kernel.

**Fig 7:**
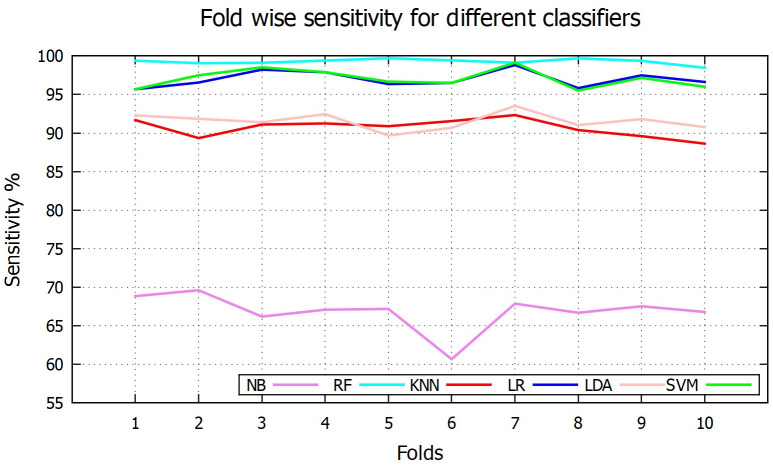
Fold wise sensitivity.

**Fig 8:**
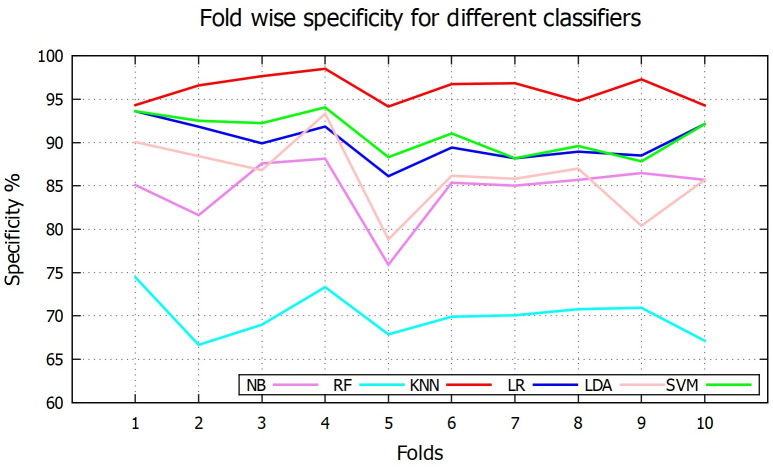
Fold wise specificity.

**Fig 9:**
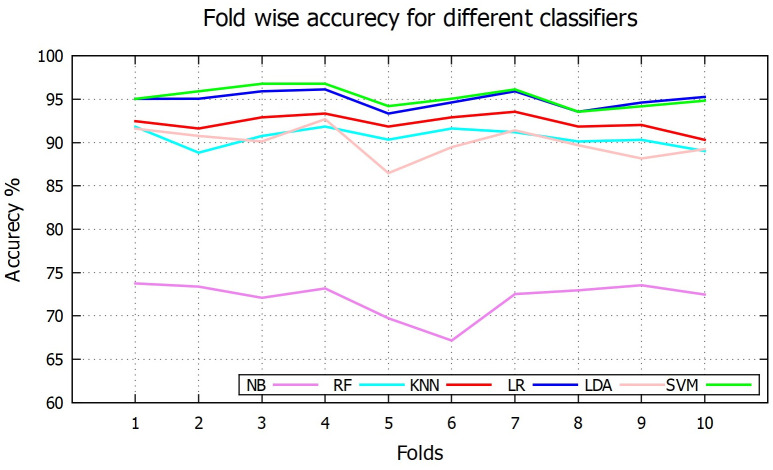
Fold wise accuracy.

Table 1 reports the overall performance for (1)–(5) of above six classifiers. Among the six classifiers, the SVM based scheme shows the overall best performance with 95.25% accuracy while the NB based method shows the overall lowest performance with 72.09% accuracy. In case of sensitivity, RF is in the highest place (99.27%), SVM in the second highest with 97.07% and NB is the lowest position (sensitivity of 66.83%). Fig 7 shows fold-wise sensitivity for the different classifiers. On the other hand, *k*NN shows the highest average specificity value of 96.13%, followed by SVM with 90.95% and RF in the last position with 70.02% specificity. Fold wise specificity for different classifiers are depicted in Fig 8. Although SVM and LR are not the best performer for individual sensitivity or specificity value but overall consideration of F1 score and accuracy values, they become the top two classifier among the others. This is because although RF has highest sensitivity value (99.27%) but very poor specificity value (70.02%). Whereas *k*NN has highest specificity value (96.13%) but low sensitivity value (90.67%) compared to others. On the other hand both SVM and LR have kept good sensitivity and specificity value to lead their F1 score and accuracy values to 0.97, 95.25% and 0.96, 94.95% respectively. Fold wise accuracy for different classifiers are given in Fig 9.

**Table 1:** Overall performance for different ML classifiers.

Classifier	Sensitivity %	Specificity %	F1 score	AUC	Accuracy %
NB	66.83	84.67	0.78	0.77	72.09
LDA	91.54	86.26	0.93	0.96	89.97
RF	**99.27**	70.02	0.94	0.97	90.59
*k*NN	90.67	**96.13**	0.94	**0.98**	92.29
LR	96.99	90.06	0.96	**0.98**	94.95
SVM	97.07	90.95	**0.97**	**0.98**	**95.25**

**Fig 10:**
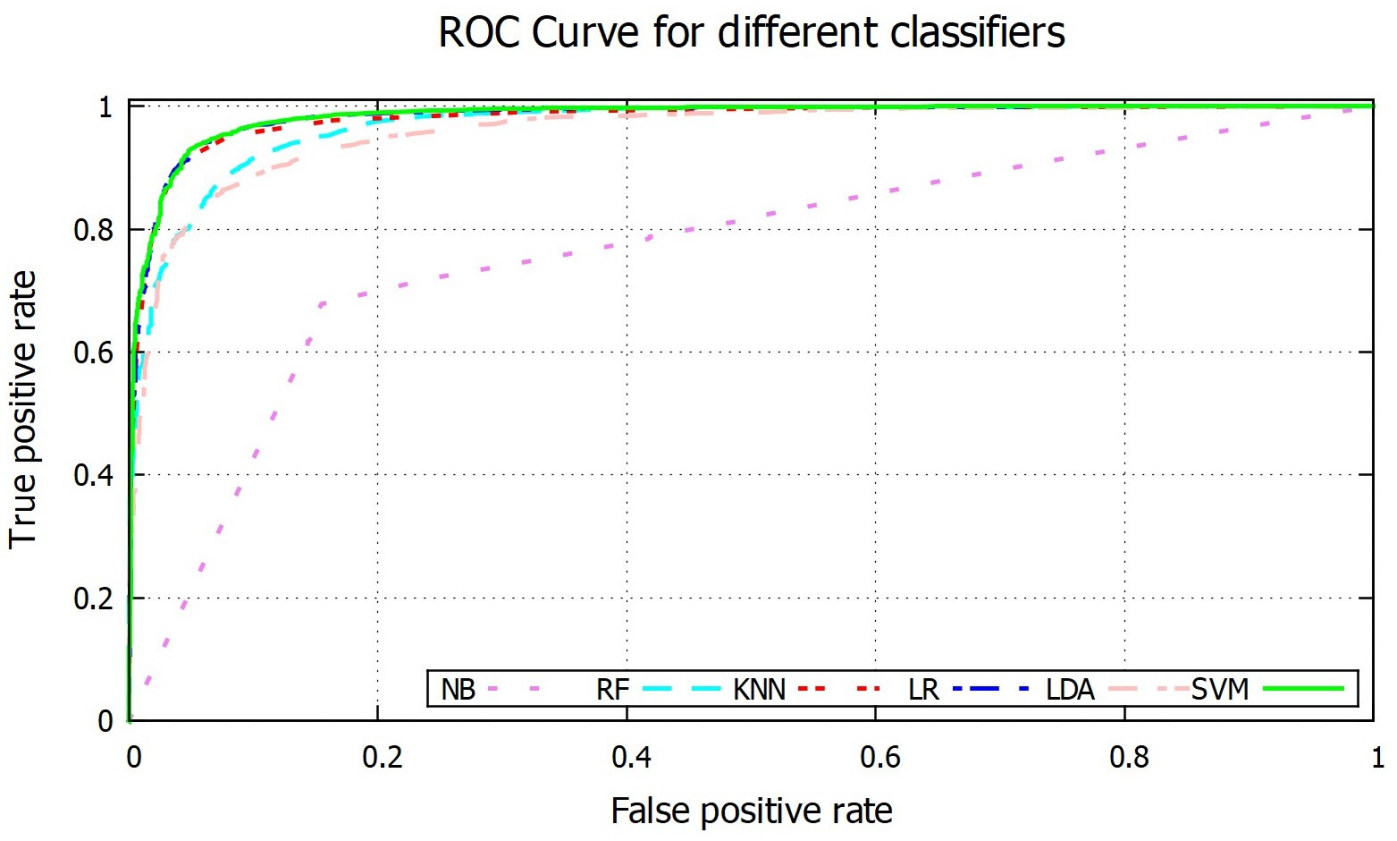
ROC graph for different ML based classifiers.

**Fig 11:**
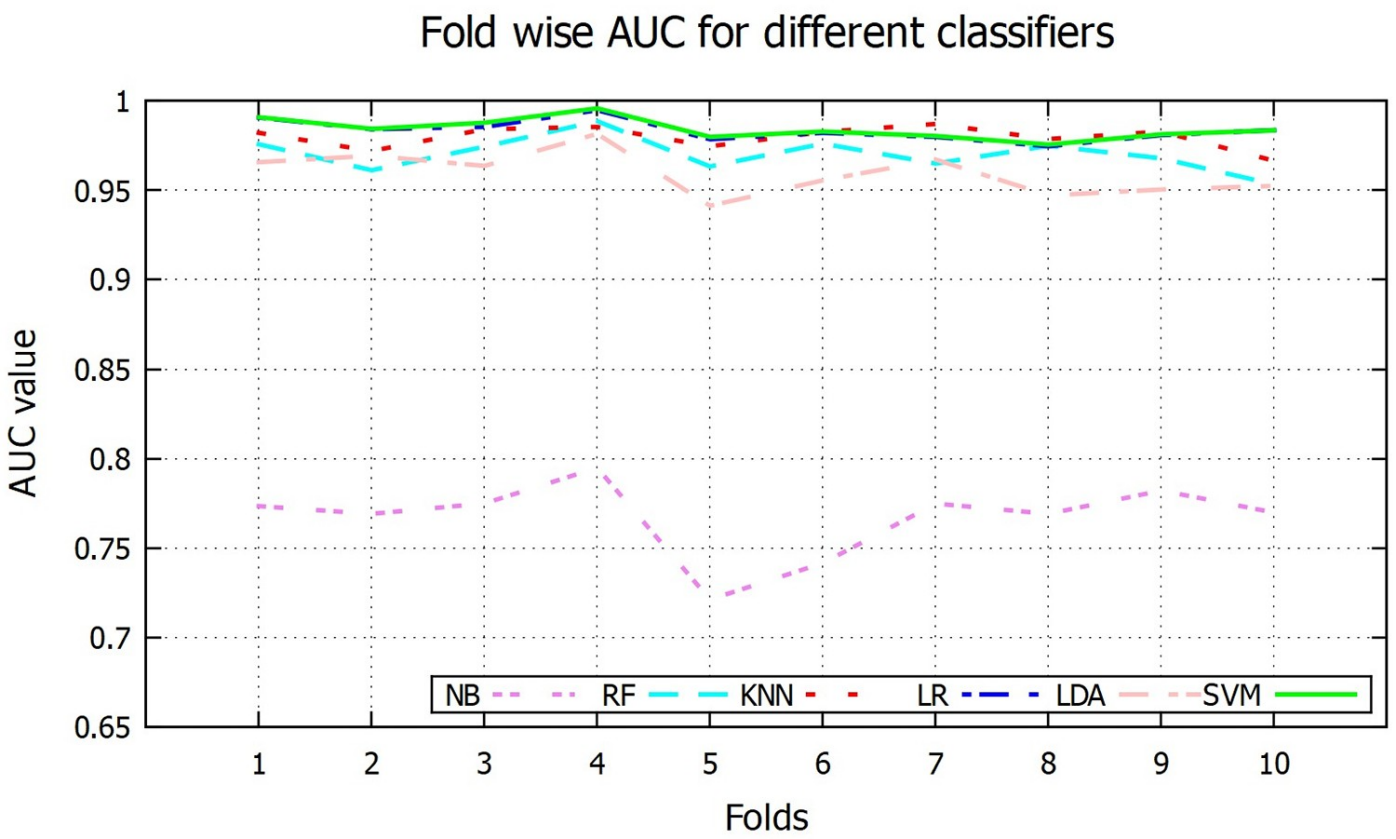
Fold wise AUC value.

In order to further evaluate the performance, ROC curve for different classifiers are shown in Fig 10 where curves of *k*NN, LR and SVM are almost overlapped. The AUC value is an index for classifier performance where lager area under ROC curve indicates better performance of the classifier. AUC values for different classifiers are given in Table 1, where SVM, LR and *k*NN have highest AUC value of 0.98 whereas NB has lowest AUC of 0.77. Fold wise AUC for different classifiers are shown in Fig 11.

### Results of deep learning based process

As mentioned in the methodology section, three different CNN models are used to perform the classification process in DL based classification. In this process, full image dataset is divided into three parts of 70%, 15%, 15%, where 70% images are used for training the model, 15% for validation and last 15% for testing the trained model. All the models are trained with 50 epochs as because the models get overfitting after that. All the models are trained using 64 batch size with additional batch size 32, 128 and 256 is used for model 3 training to check the impact of the batch size on the model’s performance. Overall performance of the different models are listed in Table 2.

**Table 2:** Overall performance for different CNN models.

Classifier	Sensitivity %	Specificity %	F1 score	AUC	Accuracy %
Model 1	98.80	89.76	0.97	0.94	96.16
Model 2	98.81	88.94	0.97	0.94	96.02
Model 3 (B:32)	97.71	**99.10**	0.99	0.98	98.15
Model 3 (B:128)	**99.60**	96.57	0.99	0.98	98.72
Model 3 (B:256)	99.39	98.12	0.99	**0.99**	99.00
Model 3 (B:64)	99.19	99.04	**1.00**	**0.99**	**99.15**

**Fig 12:**
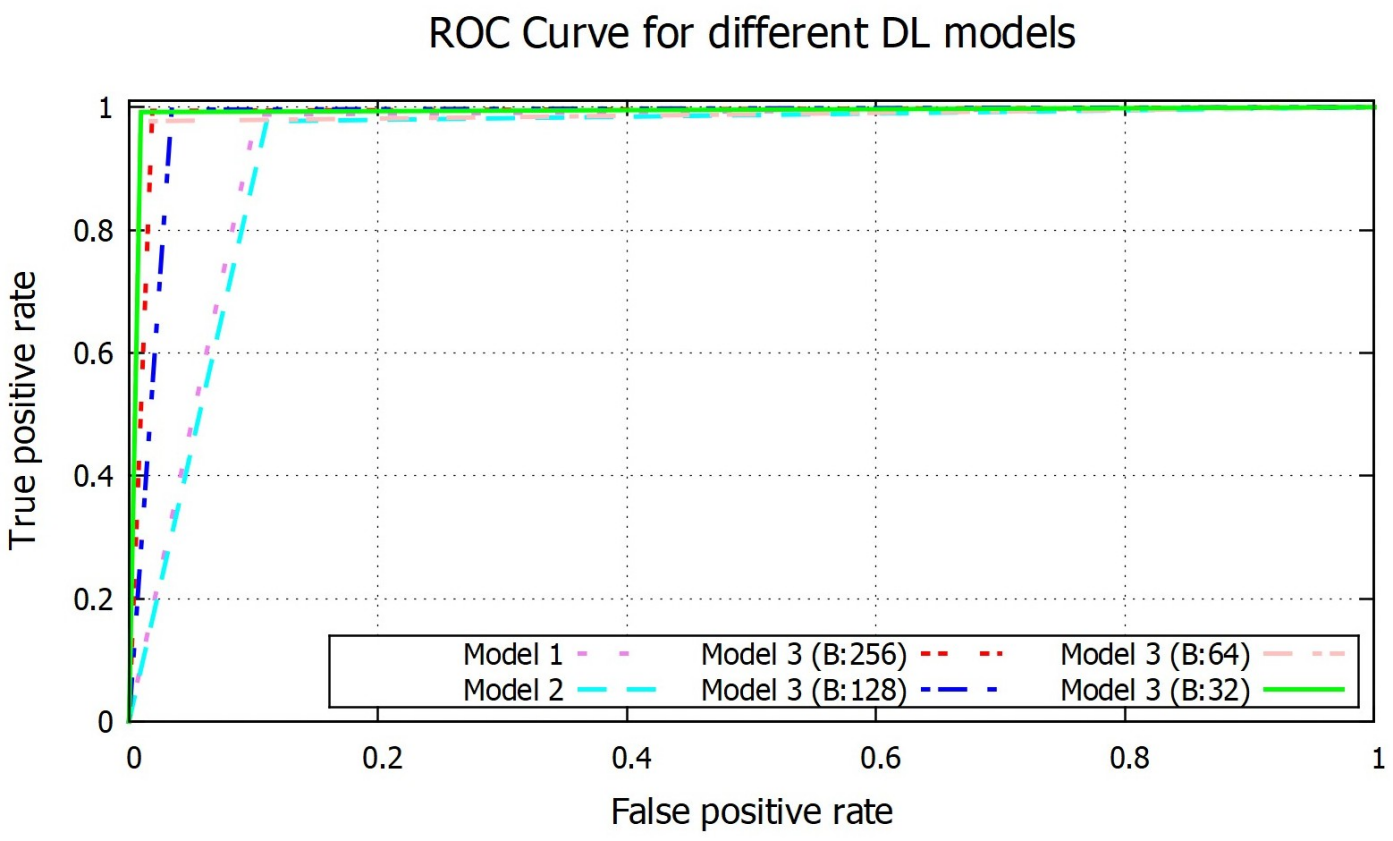
ROC graph of CNN model 3 with batch size 64.

From the Table 2, it is clear that all the DL based models give better result than the ML based classifiers. Model 3 with batch size 64 produces the best result with 99.15% accuracy and F1 score 1.00. Model 1 and 2 have good sensitivity value but due to less specificity value, overall F1 score and accuracy values are lower compared to model 3. Among the batch size based different versions of model 3, batch size 128 has produced the best sensitivity of 99.60% whereas batch size 32 gives the highest specificity value of 99.10%, but overall best performance is produced by batch size 64. Batch size 256 gives the second best overall performance with accuracy and F1 score of 99.00% and 0.99 respectively. Batch 128 and 32 are in third and fourth position with 98.72% and 98.15% accuracy respectively with both having same F1 score (0.99). ROC curve for all the DL based models are given in Fig 12. Area under ROC curve for different models are also given in Table 2, where model 3 with batch size 64 and 256 have AUC almost 1 (0.99), model 3 with batch size 32 and 128 have AUC 0.98 and model 1 and 2 have AUC 0.94.

**Fig 13:**
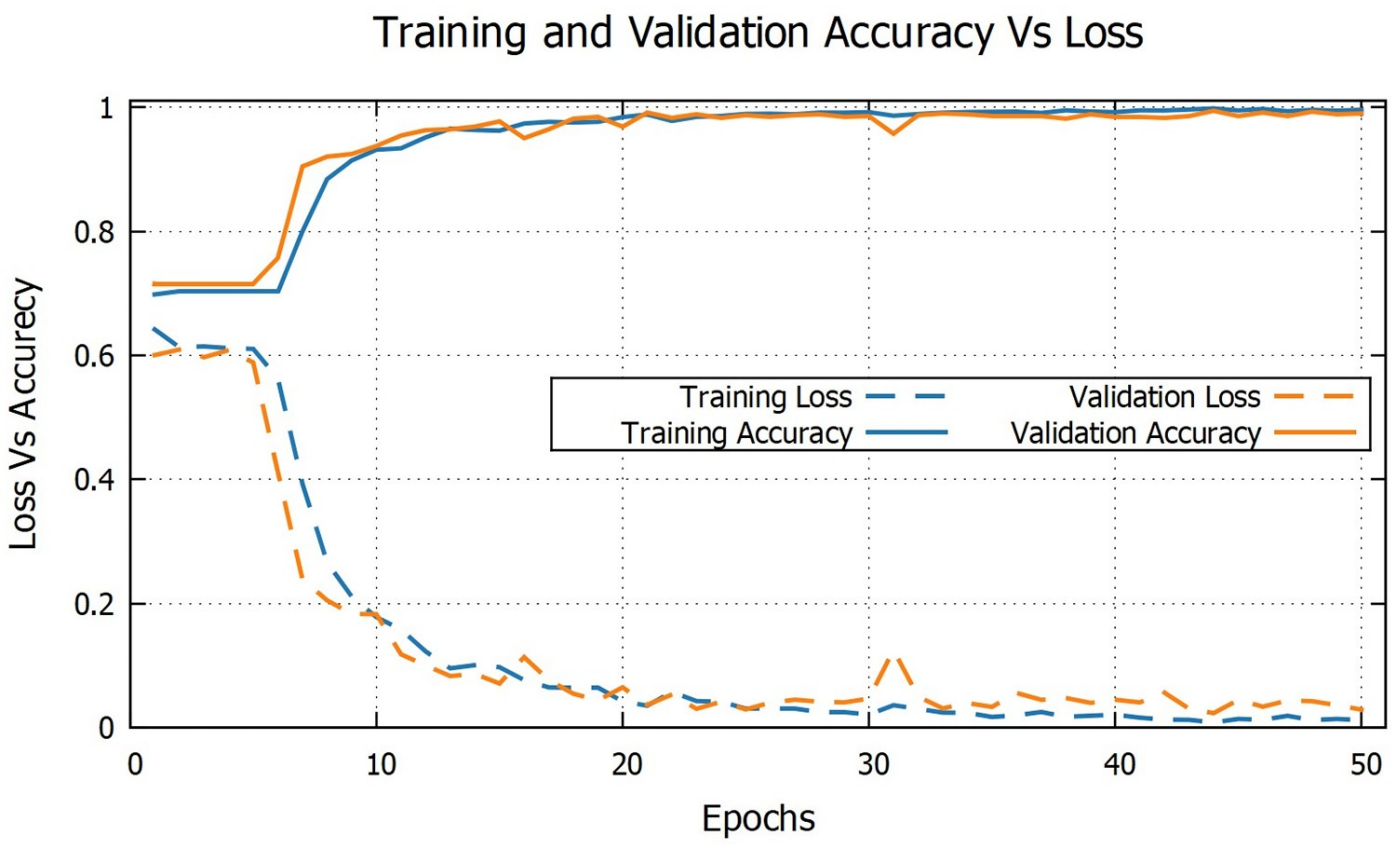
Training and validation loss Vs accuracy graph of CNN model 3 with batch size 64.

As the model 3 with batch size 64 has proven to be the best model among the proposed three DL models for identification of ASD from TD children, a detailed discussion of the loss vs accuracy graph for that model is given here. Fig 13 plots epoch wise training and validation loss vs accuracy for model 3 with batch size 64, where training and validation accuracy increases close to 100% as the losses goes down close to zero with the increase of epochs, indicating that the CNN model have found a good fit to the training data, with a lower loss value on the validation set.

The comparison of the proposed approach with existing work done using the same ASD dataset used in this analysis is shown in Table 3. Among the existing works, [39, 48, 49] used ML based classification whereas works [26, 27, 50] used DL for ASD classification.

**Table 3:** Comparison with proposed and existing methods using same dataset.

Authors	Feature extraction	Classifier	Accuracy %
Alsaggaf *et al*. [48]	FFT	FLDA	80.27
Alhaddad *et al*. [39]	FFT	FLDA	90.00
Kamel *et al*. [49]	FFT	RFLD	92.06
Nur *et al*. [50]	MLPN	MLPN	80.00
Djemal *et al*. [26]	DWT, SE	ANN	98.60
Alturki *et al*. [27]	DWT, SE	ANN	98.20
**Proposed Method**	Spectrogram image	CNN	**99.15**

In study [39, 48, 49], all researchers extracted features from EEG signal using Fast Fourier Transform (FFT) method. Using Regulated Fisher Linear Discriminant (RFLD) for classification, Kamel *et al*. [49] obtained 92.06% accuracy whereas Fisher Linear Discriminant Analysis (FLDA) was used for classification by Alsaggaf *et al*. [48] and Alhaddad *et al*. [39] to achieve an accuracy of 80.27% and 90.00% respectively. A recent study of Nur *et al*. used Multilayer Perceptron Network (MLPN) classification method and obtained an accuracy of 80% [50]. Djemal *et al*. [26] used DWT with SE on 50 seconds segment length with an ANN classifier. They achieved an accuracy of 98.60% for non-overlapping segments. Alturki *et al*. [27] also extracted features using DWT with SE and classified using ANN to obtain an accuracy of 98.20%. The proposed technique using spectrogram image with CNN classifier gives 99.15% accuracy which outperforms them all.

Table 4 provides an overall summery of existing ASD classification study using different datasets. Detailed information about those existing methods are discussed in Section. Since all those works used different dataset for validation, which makes it difficult to do a fair comparison with the proposed method to those works.

**Table 4:** Comparison with proposed and existing methods using different datasets.

Authors	Dataset	Feature extraction	Classifier	Accuracy %
Sheikhani *et al*., 2008 [11]	Own dataset	STFT	*k*NN	82.40
Ahmadlou *et al*., 2010 [23]	Iranian dataset	Wavelet and fractal dimension	RBNN	90.00
Bosl *et al*., 2011 [13]	Own dataset	mMSE	SVM	90.00
Ahmadlou *et al*., 2012 [24]	Iranian dataset	Wavelet and visibility graph	EPNN	95.50
Sheikhani *et al*., 2012 [12]	Own dataset	STFT and statistical	*k*NN	96.40
Ahmadlou *et al*., 2012 [25]	Iranian dataset	Wavelet and fuzzy logic	EPNN	95.50
Eldridge *et al*., 2014 [15]	Own dataset	SSD, mMSE	SVM, LR, NB	79.00
Grossi *et al*., 2017 [16]	Own dataset	MSROM/I-FAST	Sn, LR, SMO, *k*NN, K-CM, NB, RF	92.80
Heunis *et al*., 2018 [17]	Own dataset	RQA, PCA	SVM	92.90
Haputhanthri *et al*., 2019 [18]	Own dataset	DWT and statistical	LR, SVM, NB, RF	93.00
Jayarathna *et al*., 2019 [21]	Own dataset	statistical and entropy	RF, LR, JRip, CNN etc.	98.06
Haputhanthri *et al*., 2020 [19]	Own dataset	statistical and entropy	LR, MLP, NB, RF	88.00
Abdolzadegan *et al*., 2020 [20]	Own dataset	Linear and nonlinear	*k*NN, SVM	90.57
**Proposed Method**	**KAU**	**Spectrogram image**	**CNN**	**99.15**

## Conclusion

In this study, a T-F spectrogram image of EEG signal is introduced for classification of ASD from normal children. Short-Time Fourier transform based spectrogram images are generated from EEG signal and both the ML and DL based techniques are used for classification. For the ML based classification, six different classifiers are used to classify the features extracted by tCENTRIST. In the DL based process, three different CNN models are used on the spectrogram images. The results showed that SVM gives the highest classification accuracy in the ML based classification with an accuracy value of 95.25% and in the DL based classification process, the proposed CNN model achieved an accuracy of 99.15%. Finally, a comparison is made with other state-of-the-art approaches in the literature, that used the same dataset used in this analysis. The findings showed that with this outcome, the proposed approach outruns most of the techniques in existing literature and can be used as a basis for CAD system that aim to detect other neurological disorders where EEG recordings are used for diagnosis. In future studies, this technique can be used to develop CAD systems for other neurological disorders like Mild Cognitive Impairment and Alzheimer’s disease.

## Supporting information

S1 File (PDF)
