# Supplementary material for: A spectrogram image based intelligent technique for automatic detection of autism spectrum disorder from EEG
Source: PLoS One. 2021 Jun 25;16(6):e0253094. doi: 10.1371/journal.pone.0253094 (PMC8232415; doi:10.1371/journal.pone.0253094)
Supplement: S1 File — (PDF) [file pone.0253094.s001.pdf]

**Md. Nurul Ahad Tawhid <tawhid@iit.du.ac.bd>**

# Request for dataset

4 messages

**Md. Nurul Ahad Tawhid** <tawhid@iit.du.ac.bd> Mon, Mar 9, 2020 at 4:37 PM
To: malhaddad@kau.edu.sa

Dear Dr. Mohammed Jaffer Alhaddad,

I am Tawhid from Bangladesh. I need the datasets from the link below:

https://malhaddad.kau.edu.sa/Content-0002860-EN-214240

the request link for datasets in that page is not working, that's why I am sending you this email. So can you please send me download link for those datasets.

Thanks in advance.

Kind regards
Tawhid

---------------------------------------------------
Md. Nurul Ahad Tawhid
Assistant Professor,
Institute of Information Technology,
University of Dhaka,
Dhaka.

محمد جعفر محمد الحداد <malhaddad@kau.edu.sa> Thu, Mar 19, 2020 at 2:25 AM
To: "Md. Nurul Ahad Tawhid" <tawhid@iit.du.ac.bd>

Enjoy it

https://drive.google.com/drive/folders/0BxpGuMIiXGtHQ1BrOUxyaEg5Rnc?usp=sharing

just send me some of your information, ur name , study , university , supervisor. ,,, and please acknowledge my name and the data set in your paper.

Many thank
[Quoted text hidden]
--
Dr. Mohammed Jaffer Alhaddad
Associate Professor
Kingdom of Saudi Arabia, Jeddah 21441 P.O. Box 1828
Phone: +966 12 695 1698
King Abdulaziz University
Faculty of Computing and Information Technology
http://malhaddad.kau.edu.sa/

Disclaimer: The information contained in this message is intended for the addressee only and may contain classified information. If you are not the addressee, please delete this message and notify the sender; you should not copy or distribute this message or disclose its contents to anyone. Any views or opinions expressed in this message are those of the individual(s) and not necessarily of the university. No reliance may be placed on this message without written confirmation from an authorized representative of its contents. No guarantee is implied that this message or any attachment is virus free or has not been intercepted and amended.

إخلاء مسؤليه:  ان جميع المعلومات المتضمنة في هذه الرسالة تخص المستلم، وربما تتضمن معلومات سرية، واذا لم تكن انت المستلم فالرجاء الغاء الرسالة واشعار المرسل، كما يمنع نسخ او توزيع هذه الرسالة او افشاء محتوياتها لاخرين. ويجدر بالعلم ان جميع الآراء ووجهات النظر الواردة في هذه الرسالة تعتبر شخصية وليست بالضرورة منسوبة للجامعة. كما لايمكن ان يعتمد على هذه الرسالة ما لم تحصل على موافقة خطية من المسئول عن محتوياتها. كما لايوجد ضمان بان هذه الرسالة أو مرفقاتها خالية من الفيروسات أو انها لم يتم اعتراضها وتعديلها

---

**Md. Nurul Ahad Tawhid** <tawhid@iit.du.ac.bd> Thu, Mar 19, 2020 at 11:40 AM
To: محمد جعفر محمد الحداد <malhaddad@kau.edu.sa>

Dear Dr. Mohammed Jaffer Alhaddad,

Thank you very much for sharing the data. Details about me:

Md. Nurul Ahad Tawhid
PhD Student
Victoria University, Melbourne, Australia,
Supervisor: Prof. Hua Wang,
Co-Supervisor: Dr. Siuly

Thanks again for your data and surely I will mention you and your dataset in my papers.

With best regards,
Tawhid

[Quoted text hidden]

---

**محمد جعفر محمد الحداد** <malhaddad@kau.edu.sa> Thu, Mar 19, 2020 at 7:09 PM
To: "Md. Nurul Ahad Tawhid" <tawhid@iit.du.ac.bd>

wish you the best
[Quoted text hidden]
[Quoted text hidden]
